# Risperidone-Induced Renal Damage and Metabolic Side Effects: The Protective Effect of Resveratrol

**DOI:** 10.1155/2017/8709521

**Published:** 2017-06-15

**Authors:** Sedat Bilgiç, Deniz Taştemir Korkmaz, Sebile Azirak, Ayşe Nilay Güvenç, Nevin Kocaman, Mehmet Kaya Özer

**Affiliations:** ^1^Vocational School of Health Services, Adıyaman University, Adıyaman, Turkey; ^2^Department of Histology, Faculty of Medicine, Fırat University, Elazığ, Turkey; ^3^Department of Pharmacology, Faculty of Medicine, Adıyaman University, Adıyaman, Turkey

## Abstract

**Objective:**

The aim of the study was to investigate the possible protective qualities of resveratrol (RSV) against the side effects of risperidone (RIS) in an experimental model in rat kidneys with histologic and biochemical assessments.

**Materials and Methods:**

Experimental procedures were performed on 35 female Sprague Dawley rats. Rats were randomly divided into five groups: control, untreated rats (*n* = 7) were in group 1; group 2 was given 2 mg/kg/day RIS (*n* = 7); group 3 was treated with 2 mg/kg/day RIS and 20 mg/kg/day RSV (*n* = 7); group 4 was treated with 2 mg/kg/day RIS and 40 mg/kg/day RSV (*n* = 7); and group 5 was treated with 2 mg/kg/day RIS and 80 mg/kg/day RSV (*n* = 7). All treatments were administered for two weeks by gavage. On treatment day 15, kidney tissues were removed for analysis.

**Results:**

The results showed that RSV treatment reduced weight gain induced by RIS. In addition, RSV increased the total antioxidant status (TAS) and decreased serum creatinine (Cr), blood urea nitrogen (BUN), oxidative stress index (OSI), and total oxidant status (TOS) levels significantly (*p* < 0.05).

**Conclusion:**

This study revealed that treatment with RSV might protect kidney tissues against the side effects of RIS. RSV could be an effective course of therapy to enhance therapeutic efficacy.

## 1. Introduction

RIS, structurally, a benzisoxazole derivative, is one of the most frequently prescribed atypical antipsychotics in the management of schizophrenia, mood disorders, and autism [1]. Its mechanism of action is not entirely clear. Although the use of atypical antipsychotic drugs has been successful in the treatment of schizophrenia, they can provoke some complications, including weight gain, sedation, movement problems, sleepiness, vision difficulties, constipation, hyperprolactinemia, and extrapyramidal side effects [2]. Patients using these drugs tend to disrupt treatment primarily due to their adverse effects. In addition, RIS is a highly potent drug, extensively metabolized in the liver and excreted by the kidneys [3]. RIS and its primary active metabolite, 9-hydroxyrisperidone, are eliminated by the kidneys. In patients with moderate to severe renal disease, clearance of the sum of the parent drug and metabolite has been demonstrated to decrease by 60% compared to healthy subjects [4].

There are a few cases reporting atypical antipsychotic drug-associated renal damage in patients (including those using RIS), but the mechanism that leads to RIS-related injury is not well understood [5]. Adverse outcomes potentially attributable to these drugs, such as hypotension, acute urinary retention, and rhabdomyolysis, are known to cause this injury [6]. Moreover, pneumonia, acute myocardial infarction, and ventricular arrhythmia have been associated with these drugs in former population-based studies and renal damage may also co-occur with these events [7].

Nowadays, medicinal plants are a considerable source of drug synthesis. RSV, trans-3,5,4′-trihydroxy stilbene, has potent antioxidative and anti-inflammatory properties [8]. It is a polyphenolic phytoalexin present in many edible plants, including mulberries, peanuts, and grapes [9]. RSV has been demonstrated to exhibit a wide range of health-promoting benefits for the coronary, neurological, hepatic, and cardiovascular systems. It has been shown to inhibit inflammation, viral infection, oxidative stress, platelet aggregation, and the growth of a variety of cancer cells [10]. In addition, RSV has been studied in vivo and in vitro [11] and has been shown to possess a series of pharmacological benefits, including nephroprotective effects [12], as a result of its antioxidant and cytoprotective properties [13]. The antioxidant properties of RSV are mainly dependent upon the upregulation of endogenous cellular antioxidant systems, but the compound also displays direct reactive oxygen species (ROS) scavenging properties [14]. The high potency and low systemic toxicity of RSV make it a promising alternative to conventional therapeutic drugs. The fact that RIS exposure induces an excessive increase in metabolic alterations suggests that RSV could be used as an alternative therapy.

To our knowledge, there is no report regarding the protective and therapeutic effects of RSV on RIS-induced renal damage because it has not been studied until now. Therefore, the present study was designed to investigate the possible beneficial impact of oral supplementation with RSV against RIS-induced renal damage in rats for the first time. To achieve our goal, we performed several biochemical and histological analyses in female rats.

## 2. Materials and Methods

### 2.1. Chemicals

RIS was bought from Janssen Turkey, Istanbul, and RSV (CAS number 501-36-0) was purchased from Sigma-Aldrich (St. Louis, MO, USA). They were dissolved in distilled water. All other chemicals used were of the best analytical grade.

### 2.2. Animals

In this study, thirty-five female Sprague Dawley rats were used. Healthy adult (twelve weeks old) rats weighing 250–300 g were obtained from the Fırat University Laboratory Animal Production and Research Center. All animal care and follow-up procedures were performed at this center. The experiments were performed in accordance with the protocol approved by Fırat University Faculty of Medicine, Laboratory Animals Ethics Committee (protocol number 2016/25). The rats were kept at 21 ± 1°C for 12 h in a light-dark cycle, were fed standard rat chow, and drank tap water ad libitum. RIS and RSV were administered to the rats for two weeks.

### 2.3. Experimental Design

In our study, 35 rats were randomly assigned to five groups with an equal number in each group. We used the simple randomization technique in this experimental study. Thirty-five female Sprague Dawley rats were divided into five groups as follows: group 1, control; group 2, RIS; group 3, RIS+RSV-1; group 4, RIS+RSV-2; and group 5, RIS+RSV-3. The control group was given physiological saline solution by gavage once a day. 2 mg/kg/day RIS was administered by gavage for two weeks to all groups, except the control group. 20 mg/kg/day RSV was given for two weeks to the RIS+RSV-1 group. 40 mg/kg/day RSV was given for two weeks to the RIS+RSV-2 group. 80 mg/kg/day RSV was given for two weeks to the RIS+RSV-3 group. All compounds were suspended in physiological saline solution and administered by gavage once a day. Body weight was recorded at the beginning and end of the study. The treatment course lasted two weeks for all groups. At the end of the second week of the treatment period, the animals were euthanized by exsanguination through cardiac puncture under diethyl ether anesthesia. Before killing, rats were individually weighed, venous blood samples were collected, and serum samples for biochemical analysis were separated by centrifuge at 2800*g* for 15 min and then stored at −80°C. Kidneys were surgically removed, weighed, and stored at −80°C for subsequent biochemical and pathohistological analysis.

The daily dose of RIS varies between 0.25 and 16 mg, and a frequently prescribed dose in previous studies was 2 mg [15]. The maximum recommended human dose (MRHD) of RIS is 0.4–12 mg/day. The experimental dose was calibrated as 2.0 mg/kg (MHRD × 10) on the basis of per kg body weight per day and its suitability to the rat animal model [16]. Therefore, a 2 mg dose was selected for the present study. The assigned dosage of powdered RIS (2 mg/kg once a day for two weeks) was administered by a gastric tube daily between 8:00 and 9:00 a.m. in line with a previous report [17]. RSV (20, 40, and 80 mg/kg body weight/day) was also administered by a gastric tube daily between 8:00 and 9:00 a.m. The dose and duration of RSV were selected according to results from a previous study [18].

### 2.4. Biochemical Analysis

BUN was measured using a commercially available enzymatic colorimetric method and analyzer system (Hitachi 917 modular device; Roche Diagnostics, Basel, Switzerland). Serum Cr was measured by Jaffe's method using the same analyzer system [19].

### 2.5. TAS and TOS Determination

The automated calorimetric measurement methods developed by Erel were used to define the TAS (mmol/L) and TOS (*μ*mol/L) (a serum oxidant parameter). The measurement of TAS and TOS in serum samples was determined by a TAS and TOS kit (REL Assay Diagnostics) [20, 21].

### 2.6. OSI Determination

OSI was determined as TOS to TAS ratio and was calculated as follows: OSI (arbitrary unit) = ((TOS, *μ*mol H2O2 eq/L)/(TAS, *μ*mol Trolox eq/L)) [22].

### 2.7. Terminal Deoxynucleotidyl Transferase dUTP Nick End Labeling (TUNEL) Assay

Apoptotic cells were defined using the ApopTag Plus Peroxidase In Situ Apoptosis Detection Kit (Chemicon, cat. number: S7101, USA) according to the manufacturer's instructions. Sections (5 *μ*m) taken from the paraffin blocks were entrenched onto polylysine-coated slides, deparaffinized using xylene, dehydrated with a series of alcohol rinses, and then washed with phosphate-buffered saline (PBS). Then, tissues were incubated with a proteinase K solution (0.05%) and with 3% hydrogen peroxide for 5 min to forestall endogenous peroxidase activity. After washing with PBS, the tissues were incubated with Equilibration Buffer for 6 min and in working solution (70% *μ*L reaction buffer + 30% TdT enzyme) at 37°C under moist conditions for 60 min. Tissues were then incubated in stop/wash buffer for 10 min and incubated in anti-digoxigenin-peroxidase for 30 min. Apoptotic cells were examined using the diaminobenzidine substrate. Cross sections contrast stained with methyl green were sealed using a proper covering solution. Mamma tissue was used as a positive control. PBS was used instead of the TdT enzyme in the negative control tissue. Preparations were examined and assessed using a research microscope (Olympus BH2 light microscope) and then photographed. To assess the TUNEL staining, after staining with methyl, green cells with green nuclei were considered normal whereas cells with brown nuclei were considered apoptotic. Apoptotic (TUNEL positive) cells were counted and statistically assessed. This analysis was made in at least eight areas of each kidney section (two sections/animal), and the sections were analyzed at 400x magnification [23].

The evaluation of TUNEL staining was made based on the extent of the staining of apoptotic cells. The extent of TUNEL staining was scored semiquantitatively as 0 (none), 1 (light), 2 (medium), and 3 (intense) [24].

### 2.8. Statistical Analyses

Statistical analysis was performed using SPSS 16.0 (SPSS, Chicago, IL, USA). All data were expressed as mean values ± their standard errors (SEM). Normality for variables in the groups was determined by the Shapiro-Wilk test. For the comparison of the mean weight of all groups, a paired *t*-test was performed. The groups were compared with the paired-samples *t*-test at the beginning and end of the treatment. One-way analysis of variance (ANOVA) followed by the LSD post hoc test were used for the comparison of biochemical parameters and total oxidant/antioxidant levels. Significance was considered at the *p* < 0.05 level. For histopathological analysis, results were expressed as the means ± standard deviation (SD). The statistically significant difference was determined by ANOVA followed by Tukey's multiple comparison test. Probability values (*p*) less than 0.05 were considered to be statistically significant.

## 3. Results

### 3.1. Effects of RIS and RSV on Weight Gain/Loss

Body weight measurements showed that during the two weeks, animals grew from 238.28 g at day one to 252.85 g for the control group, from 234.57 g at day one to 248.00 g for the RIS group, from 225.28 g at day one to 233.71 g for the RIS+RSV-1 group, from 232.40 g at day one to 226.80 g for the RIS+RSV-2 group, and from 244.80 g at day one to 246.80 g for the RIS+RSV-3 group at day 14 (Table 1: paired-samples *t*-test for the body weight at day 14; *p* = 0.000, *p* = 0.005, *p* = 0.005, *p* = 0.071, and *p* = 0.537, resp.).

**Figure 1:**
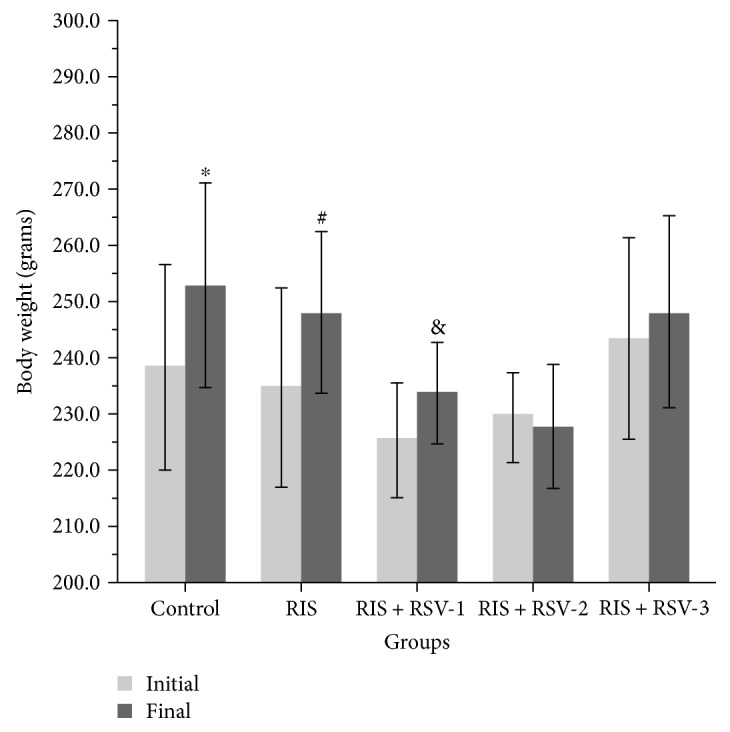
Changes in the body weight of experimental rats. Values are expressed as mean ± SEM of seven animals. The groups were compared with the paired-samples *t*-test at initial and final treatment. ^∗, #, &^In each column, different superscript characters mean significant differences at *p* < 0.05 in different groups. Abbreviations: RIS, risperidone; RSV, resveratrol; RIS+RSV-1, 2 mg/kg RIS+20 mg/kg RSV; RIS+RSV-2, 2 mg/kg RIS+40 mg/kg RSV; RIS+RSV-3, 2 mg/kg RIS+80 mg/kg RSV.

There was a significantly increased total body weight gain in the control, RIS, and RIS+RSV-1 treatment groups (*p* = 0.000, *p* = 0.005, and *p* = 0.005, resp.). However, the RIS+RSV-2 group was observed to have a decreased weight gain and the RIS+RSV-3 group an increased weight gain had no significant effect on these measurements (*p* = 0.071, *p* = 0.537, resp.) (Table 1; Figure 1).

### 3.2. Effects of RIS and RSV on Biochemical and Oxidative Stress Parameters

**Figure 2:**
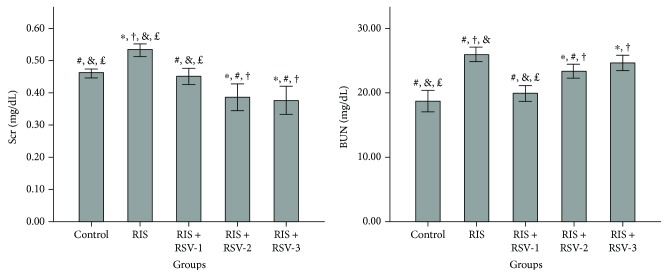
Effects of risperidone, resveratrol, and their coadministration on the kidney level of serum creatinine and blood urea nitrogen in rats after two weeks. Values are expressed as mean ± SEM of seven animals. ANOVA followed by the LSD post hoc test were used. ^∗^*p* < 0.05 versus control; ^#^*p* < 0.05 versus RIS-treated rats; ^†^*p* < 0.05 versus RIS+RSV-1-treated rats; ^&^*p* < 0.05 versus RIS+RSV-2-treated rats; and ^£^*p* < 0.05 versus RIS+RSV-3-treated rats. Abbreviations: RIS, risperidone; RSV, resveratrol; Scr, serum creatinine; BUN, blood urea nitrogen; RIS+RSV-1, 2 mg/kg RIS+20 mg/kg RSV; RIS+RSV-2, 2 mg/kg RIS+40 mg/kg RSV; RIS+RSV-3, 2 mg/kg RIS+80 mg/kg RSV.

We measured levels of biochemical parameters in serum, the results are shown in Table 2, and oxidative stress parameters are shown in Table 3. Serum Cr level was significantly increased in the RIS group compared to the control, RIS+RSV-1, RIS+RSV-2, and RIS+RSV-3 groups (*p* < 0.00). Serum Cr level was significantly increased in the RIS+RSV-1 group compared to the RIS+RSV-2 and RIS+RSV-3 groups (*p* = 0.01, *p* = 0.00, resp.) (Table 2; Figure 2).

BUN level was significantly increased in the RIS group compared to the control, RIS+RSV-1, and RIS+RSV-2 groups (*p* = 0.000, *p* = 0.000, and *p* = 0.001, resp.). BUN level was significantly decreased in the RIS+RSV-1 group compared to the RIS, RIS+RSV-2, and RIS+RSV-3 groups (*p* = 0.000, *p* = 0.000, and *p* = 0.000, resp.) (Table 2; Figure 2).

**Figure 3:**
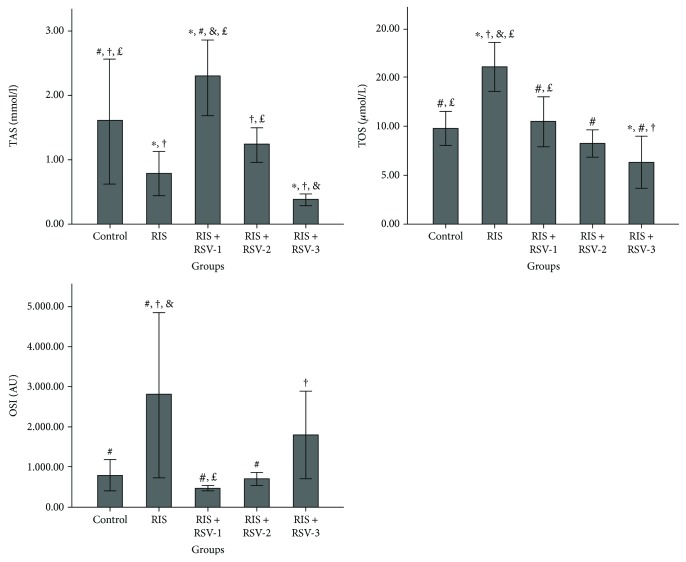
Effects of risperidone, resveratrol, and their coadministration on the level of TAS, TOS, and OSI in rats after two weeks. Values are expressed as mean ± SEM of seven animals. ANOVA followed by the LSD post hoc test were used. ^∗^*p* < 0.05 versus control; ^#^*p* < 0.05 versus RIS-treated rats; ^†^*p* < 0.05 versus RIS+RSV-1-treated rats; ^&^*p* < 0.05 versus RIS+RSV-2-treated rats; and ^£^*p* < 0.05 versus RIS+RSV-3-treated rats. Abbreviations: RIS, risperidone; RSV, resveratrol; TAS, total antioxidant status; TOS, total oxidant status; OSI, oxidative stress index; RIS+RSV-1, 2 mg/kg RIS+20 mg/kg RSV; RIS+RSV-2, 2 mg/kg RIS+40 mg/kg RSV; RIS+RSV-3, 2 mg/kg RIS+80 mg/kg RSV; AU: arbitrary units.

Ameliorative effects of RSV treatment against RIS administration significantly increased the TAS level and decreased TOS and OSI levels (*p* < 0.05). The control group had a significantly higher TAS level compared to the RIS group (*p* = 0.014). The RIS+RSV-1 group had a significantly higher TAS level compared to the control, RIS, RIS+RSV-2, and RIS+RSV-3 groups (*p* = 0.037, *p* = 0.000, *p* = 0.002, and *p* = 0.000, resp.). The RIS+RSV-2 group had a significantly higher TAS level compared to the RIS+RSV-3 groups (*p* = 0.011). TOS level was significantly higher in the RIS group compared to the control, RIS+RSV-1, RIS+RSV-2, and RIS+RSV-3 groups (*p* < 0.001). The RIS+RSV-1 group had a significantly higher TOS level compared to the RIS+RSV-3 group (*p* = 0.003). OSI level was significantly higher in the RIS group compared to the control, RIS+RSV-1, and RIS+RSV-2 groups (*p* = 0.003, *p* = 0.001, and *p* = 0.002, resp.) (Table 3; Figure 3).

### 3.3. Evaluation of Apoptosis in Kidney Tissues

**Figure 4:**
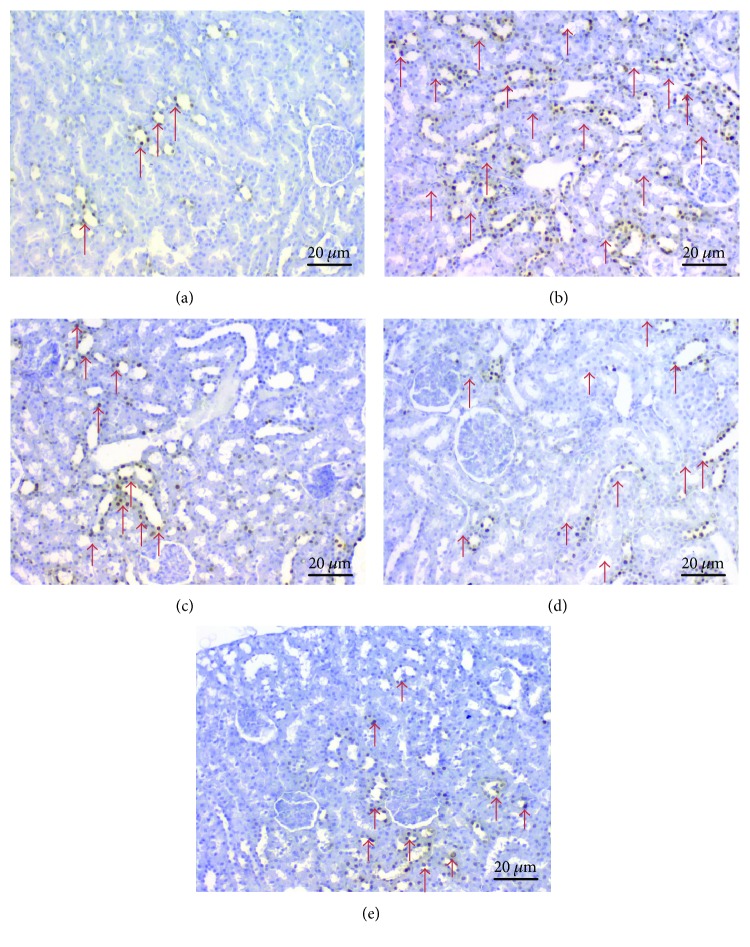
Representative photomicrographs of TUNEL staining in all five groups (scale bars = 20 *μ*m). (a) Group 1 (control) only few TUNEL-positive cells (arrow). (b) Group 2 (RIS) a lot of TUNEL-positive cells (arrows). (c) Group 3 (RIS+RSV-1), (d) group 4 (RIS+RSV-2), and (e) group 5 (RIS+RSV-3) similarly rare TUNEL-positive cells (arrows). This analysis was exerted in at least eight areas of each kidney section (two sections/animal), and the sections were analyzed at 400x magnification. The evaluation of TUNEL staining was exerted based on the extent of the staining of apoptotic cells. The extent of TUNEL staining was scored semiquantitatively as 0 (none), 1 (light), 2 (medium), and 3 (intense).

The results of the apoptotic index are shown in Table 4 and Figure 4. Using TUNEL assay to detect apoptotic renal tubular cells in the kidney sections, the control group (Figure 4(a)) showed only a few TUNEL-positive cells. The number of TUNEL-positive cells markedly increased in the RIS group (Figure 4(b)) compared with the control group (*p* < 0.05). RIS+RSV-1 (Figure 4(c)), RIS+RSV-2 (Figure 4(d)), and RIS+RSV-3 (Figure 4(e)) groups were similar and showed rare TUNEL-positive cells. Treatment with RSV (RIS+RSV-1, RIS+RSV-2, and RIS+RSV-3 groups) (Figures 4(c), 4(d), and 4(e)) reduced the number of TUNEL-positive cells as compared with the RIS group (*p* < 0.05).

## 4. Discussion

Certain herbal medicines have been declared to be effective in the treatment of adverse outcomes attributed to atypical antipsychotic drugs, including RIS, and combination treatment with drugs and herbal medicines has been reported to be useful in enhancing treatment influence and reducing recovery time and adverse effects. In the present study, RIS (2 mg/kg/day) plus RSV (20, 40 and 80 mg/kg/day) was given by gavage to rats for two weeks. All doses of RSV caused dose-dependent decreases in weight gain and alleviated renal damage of the animals compared to the group that received only RIS. Thus, the present study aimed to investigate whether RSV implemented a protective effect on RIS-induced renal damage and metabolic side effects in an experimental animal model.

Second-generation antipsychotics (SGAs) are extensively used in several psychiatric disease entities and exert to various extents the risk of antipsychotic-induced weight gain. In a study by Domecq et al., weight gain was associated with the use of RIS [25]. A meta-analysis by Allison et al. in adults estimated that the mean weight gain after 10 weeks of treatment was 2.00 kg for RIS [26]. Weight gain can impair both physical health and psychological well-being. Therefore, it is important to define factors that are associated with the risk of weight gain with atypical antipsychotics. These factors may include lifestyle issues, particularly diet. RSV, being a dietary constituent, is an excellent therapy candidate for disorders with a metabolic origin. Since several studies using animal models of diet-induced obesity have displayed the beneficial effects of RSV on reducing obesity, many clinical trials have been effected to assess its effect in humans. In a study by Gómez-Zorita et al., RSV decreased whole body weight in obese rats by reducing oxidative stress [27]. Dal-Pan et al. postulated resveratrol's facilitation of weight loss in nonhuman primate models of obesity by increasing the metabolic rate and suppressing torpor expression. In fact, RSV administration in primate models led to a 13% reduction in energy intake while increasing the resting metabolic rate by 29% [28]. In the current study, RSV cotreatment diminished antipsychotic-induced weight gain with a more obvious effect and significantly decreased only with a 20 mg/kg dose. These results imply that the mechanism of action for RSV occurs by increasing energy expenditure, directly reducing energy storage and inhibition of energy intake. This weight decrement effect of RSV is estimated to be in part attributable to its effects on adipocytes [29, 30]. Hence, RSV, a dietary supplement, significantly prevents RIS-induced weight gain, which might suggest a potential effectiveness on human subjects. Therefore, RSV is a safe compound for coadministration with RIS for mitigating antipsychotic-induced weight gain/obesity without influencing their therapeutic action. In addition, these results are certain to influence the choice of protective compound when several options exist and to institute preemptive strategies for weight management.

Several adverse outcomes attributed to atypical antipsychotic drugs are known to cause renal damage. RIS was also described as increasing the risk of this damage in a population study [31]. Serum Cr and BUN were the classical standards to evaluate renal damage [32]. Accordingly, a study conducted by Hsu et al. demonstrated that elevated serum Cr was observed in RIS administration [33]. Therefore, the study of Hsu et al. is an indicator to prove renal damage through the use of RIS. RIS may impair tubular function and result in several renal complications. As a polyphenolic phytoalexin, RSV has been reported to be useful in the prevention of numerous types of kidney disease [34, 35], drug-induced renal damage [12], and ischemia-reperfusion and sepsis-induced kidney injuries [36]. Similarly, Wu et al. demonstrated that RSV effectively attenuated renal oxidative stress in the diabetic rat kidney [37]. A number of independent studies further explored the molecular mechanisms of RSV-mediated nephroprotection. The results of the present study demonstrate that RIS exposure produced a significant increase in the level of serum Cr and BUN indicating damaged structural and functional kidney integrity. Consistent with these observations, the beneficial effects of RSV were identified in the prevention of renal tubular damage and dysfunction. In addition, oral supplementation of RSV demonstrated the restoration of the elevated serum Cr and BUN to the normal levels [38, 39]. RSV cotreatment ameliorated these changes in all doses, with an especially obvious effect in high doses. These results are supported by data in the literature [40].

Oxidative stress is one of the key mechanisms responsible for renal damage and disease progression. Antioxidants try to fight oxidative stress and minimize its damage. TAS measurement has been used to evaluate the overall performance of the antioxidant system. TOS measurement provides a sensitive index of lipid peroxidation and oxidative stress [41]. The TOS/TAS ratio is termed “OSI,” which is an indicator of the oxidative stress degree [42]. In the present study, RIS administration resulted in a decrease in TAS level and an increase in TOS and OSI levels as in former studies [43]. Conversely, we observed that RSV could protect kidneys from RIS-induced renal damage and provide useful changes in the basal levels of stress biomarkers. We found that the serum TAS level increased and the TOS and OSI levels prominently decreased with RSV treatment as reported in previous studies [44]. Similarly, Wong et al. demonstrated that RSV reduced oxidative damage biomarkers during aging in F2 hybrid mice [45]. In addition, RSV cotreatment ameliorated these changes with a more obvious effect in 20 mg/kg doses. The beneficial effects of RSV, observed in the current work, are very likely due to its strong antioxidant properties and may be associated with its constituent compounds. This implies that the RSV presented a nephroprotective activity probably due to its antioxidant capacity. The restoration of tissue antioxidant function by RSV may be attributed to its ability to upregulate antioxidant gene expression. Also, RSV may display its antinephrolithic properties by both inhibiting free radical formation and attenuating the expression of a number of inflammatory mediators [46].

Histological observations added more evidence of the protective effect of RSV. Apoptosis involves interactions among several protein families that regulate activation of various apoptotic markers. More recently, Elmorsy et al. demonstrated that some antipsychotics, including RIS, can induce apoptosis [47]. Apoptosis effects triggered by RIS appear to be cell-type dependent [48]. ROS can also induce cell death via apoptosis in many cell types. Such an effect was also blocked by RSV [49]. RSV is accepted as a potent antioxidant and antiapoptotic agent [50, 51]. In the present study, histopathological evaluation of the kidneys showed severe damage ensued through loss of normal architecture, which included vacuolar degeneration and fatty changes in RIS-administered rats. These toxic effects were effectively prevented by RSV treatment. Furthermore, RSV cotreatment ameliorated these changes with a more obvious effect in 80 mg/kg doses. The antiapoptotic effect of RSV based on the evaluated reduction in TUNEL-positive kidney cells may also contribute to its therapeutic impact against RIS. Therefore, the antioxidant activity of this compound may be a potential mechanism for antiapoptotic activity. Accordingly, RSV treatment of the cells against RIS exposure prevented apoptotic cell injury and death. The underlying mechanism of protection of RSV may be associated with the suppression of apoptosis via death receptor-mediated pathways. On the other hand, because the kidneys contain more mitochondria than the other organs, the nephrotoxic effects might also be due to the direct action of RIS on renal mitochondria. Therefore, the reducing effects of RIS on mitochondrial functions may also be important in the pathogenesis of nephrotoxicity [52, 53]. Hence, it can be assumed that the antioxidant activity of RSV may be due to the effect on the mitochondria-dependent apoptotic pathway, but this has to be supported with further experimentation. Therefore, RSV may be a good option against RIS-induced side effects. The mechanism of action of RIS has not been fully explained, and its exact renal damage mechanism is still being researched because of insufficient experimental study in the literature histopathologically investigating the effects of RIS on renal cells in rats.

RSV was revealed to facilitate a significant reduction in serum Cr and BUN levels and to alleviate renal damage and dysfunction, which was further verified by apoptosis analysis of renal histology. In addition, RSV reduced weight gain, contributed to the oxygen radical scavenging activity, and increased antioxidant activity to accomplish its protective ability against damage caused by the side effects of RIS. Therefore, RSV may act through a range of mechanisms whose effects might have major therapeutic potential in RIS-induced side effects.

In conclusion, our results clearly indicate that RSV oral supplementation, in certain doses, protects against RIS-induced renal damage. Therefore, RSV may be a clinically promising agent in RIS-induced renal damage and its metabolic side effects. Further studies should be undertaken to examine the potential effect of RSV on renal damage in human and animal models.

## Figures and Tables

**Table 1:** Body weight (g) of animals during treatment.

Design of treatment	Control	RIS	RIS+RSV-1	RIS+RSV-2	RIS+RSV-3
Initial study	238.28 ± 7.53	234.57 ± 7.27	225.28 ± 4.16	232.40 ± 3.55	244.80 ± 9.88
Final study	252.85 ± 7.46	248.00 ± 5.86	233.71 ± 3.68	226.80 ± 5.25	246.80 ± 9.42
Statistical comparison (initial study versus final study)			(*p*)		
Control			**0.000**		
RIS			**0.005**		
RIS+RSV-1			**0.005**		
RIS+RSV-2			**0.071**		
RIS+RSV-3			**0.537**		

Changes in the body weight of experimental rats. Values are expressed as mean ± SEM of seven animals. The groups were compared with the paired-samples *t*-test at initial and final treatment. *p* ≤ 0.05. RIS: risperidone; RSV: resveratrol; RIS+RSV-1: 2 mg/kg RIS+20 mg/kg RSV; RIS+RSV-2: 2 mg/kg RIS+40 mg/kg RSV; RIS+RSV-3: 2 mg/kg RIS+80 mg/kg RSV.

**Table 2:** Levels of serum biochemical parameters for all groups.

Parameters	Control	RIS	RIS+RSV-1	RIS+RSV-2	RIS+RSV-3	*p*
Scr (mg/dL)	0.46 ± 0.00^b,d,e^	0.53 ± 0.00^a,c,d,e^	0.45 ± 0.01^b,d,e^	0.38 ± 0.01^a,b,c^	0.37 ± 0.01^a,b,c^	**0.000**
BUN (mg/dL)	18.71 ± 0.68^b,d,e^	25.85 ± 0.45^a,c,d^	19.85 ± 0.50^b,d,e^	23.28 ± 0.42^a,b,c^	24.57 ± 0.48^a,c^	**0.000**

Each group represents the mean ± SEM for seven rats. ^a^*p* < 0.01 versus the control group; ^b^*p* < 0.01 versus the RIS group; ^c^*p* < 0.01 versus the RIS+RSV-1 group; ^d^*p* < 0.01 versus the RIS+RSV-2 group; and ^e^*p* < 0.01 versus the RIS+RSV-3 group. RIS: risperidone; RSV: resveratrol; Scr: serum creatinine; BUN: blood urea nitrogen; RIS+RSV-1: 2 mg/kg RIS+20 mg/kg RSV; RIS+RSV-2: 2 mg/kg RIS+40 mg/kg RSV; RIS+RSV-3: 2 mg/kg RIS+80 mg/kg RSV.

**Table 3:** Comparison of serum oxidative stress parameters among the groups.

Parameters	Control	RIS	RIS+RSV-1	RIS+RSV-2	RIS+RSV-3	*p*
TOS (*μ*mol/L)	9.74 ± 0.71^b,e^	16.04 ± 1.03^a,c,d,e^	10.45 ± 1.06^b,e^	8.19 ± 0.56^b^	6.27 ± 1.09^a,b,c^	**0.000**
TAS (mmol/L)	1.60 ± 0.39^b,c,e^	0.78 ± 0.14^a,c^	2.28 ± 0.23^a,b,d,e^	1.23 ± 0.10^c,e^	0.38 ± 0.03^a,c,d^	**0.000**
OSI (AU)	791.59 ± 162.39^b^	2805.78 ± 857.32^a,c,d^	464.05 ± 25.47^b,e^	692.31 ± 69.32^b^	1797.28 ± 452.35^c^	**0.004**

Each group represents the mean ± SEM for seven rats. ^a^*p* < 0.04 versus the control group; ^b^*p* < 0.02 versus the RIS group; ^c^*p* < 0.05 versus the RIS+RSV-1 group; ^d^*p* < 0.03 versus the RIS+RSV-2 group; and ^e^*p* < 0.05 versus the RIS+RSV-3 group. RIS: risperidone; RSV: resveratrol; TAS: total antioxidant status; TOS: total oxidant status; OSI: oxidative stress index; RIS+RSV-1: 2 mg/kg RIS+20 mg/kg RSV; RIS+RSV-2: 2 mg/kg RIS+40 mg/kg RSV; RIS+RSV-3: 2 mg/kg RIS+80 mg/kg RSV; AU: arbitrary units.

**Table 4:** Effects of risperidone and resveratrol on apoptotic index (%) in rat kidneys.

Groups	Apoptotic index (%) (AI; mean ± SD)
Control	4.83 ± 1.17^b,c,d,e^
RIS	32.50 ± 3.56^a,c,d,e^
RIS+RSV-1	14.16 ± 2.40^a,b^
RIS+RSV-2	14.20 ± 2.86^a,b^
RIS+RSV-3	12.00 ± 2.60^a,b^

The apoptotic index of all the groups. Values are mean ± SD for seven rats in each group. ^a^Significant from control; ^b^Significant from RIS; ^c^Significant from RIS+RSV-1; ^d^Significant from RIS+RSV-2; and ^e^Significant from RIS+RSV-3 (*p* ≤ 0.05). RIS: risperidone; RSV: resveratrol; RIS+RSV-1: 2 mg/kg RIS+20 mg/kg RSV; RIS+RSV-2: 2 mg/kg RIS+40 mg/kg RSV; RIS+RSV-3: 2 mg/kg RIS+80 mg/kg RSV. The extent of TUNEL staining was scored semiquantitatively as 0 (none), 1 (light), 2 (medium), and 3 (intense).

## Abbreviations

BUN: Blood urea nitrogen
MRHD: Maximum recommended human dose
OSI: Oxidative stress index
PBS: Phosphate-buffered saline
RIS: Risperidone
ROS: Reactive oxygen species
RSV: Resveratrol
Cr: Creatinine
SGAs: Second-generation antipsychotic drugs
TAS: Total antioxidant status
TOS: Total oxidant status
TUNEL: Terminal deoxynucleotidyl transferase dUTP nick end labeling.
